# Classroom climate dimensions, self-efficacy, and music aesthetic literacy: a mediation study with Chinese non-music major college students

**DOI:** 10.3389/fpsyg.2025.1716470

**Published:** 2026-01-13

**Authors:** Liangliang Zhao, Wei Huang, Bo Han, Xiandi Wu

**Affiliations:** 1 Teacher School of Education, Chongqing Three Gorges University, Chongqing, China; 2 Faculty of Social Sciences and Liberal Arts, UCSI University, Kuala Lumpur, Malaysia; 3 College of Arts, Chongqing Three Gorges University, Chongqing, China

**Keywords:** classroom climate, general music education, music aesthetic literacy, physical environment, teacher-student interactions

## Abstract

**Objective:**

This study investigates the mechanisms influencing music aesthetic literacy (MAL) among Chinese non-music-major university students, focusing on the direct effects of a multidimensional classroom climate and the mediating role of self-efficacy (SE), based on Bandura’s social cognitive theory.

**Methods:**

A cross-sectional survey was conducted with 505 students from universities in Chongqing, China. Data were collected using adapted scales measuring classroom climate (physical environment-PE, teacher-student interaction-TSI, peer relationships-PR, teachers’ orientation toward learning-TOTL), self-efficacy, and music aesthetic literacy. Partial least squares structural equation modeling (PLS-SEM) was employed to test the hypothesized direct and mediating effects.

**Results:**

Findings revealed significant direct effects of all four classroom climate dimensions on MAL, with TOTL exhibiting the strongest direct effect (*β* = 0.264, *p* < 0.001). Crucially, self-efficacy partially mediated the relationships between each climate dimension and MAL, with significant indirect effects (e.g., TSI → SE → MAL: *β* = 0.047, *p* = 0.005). Teacher-student interaction was the strongest predictor of self-efficacy (*β* = 0.310, *p* < 0.001). The model explained substantial variance in both self-efficacy (*R*^2^ = 0.439) and music aesthetic literacy (*R*^2^ = 0.553).

**Conclusion:**

The results elucidate a “classroom climate – self-efficacy – aesthetic literacy” mechanism, demonstrating that a supportive classroom environment enhances students’ music aesthetic literacy both directly and indirectly by fostering their confidence. This study provides empirical evidence for educators and policymakers to optimize classroom environments and integrate self-efficacy development as a core component in aesthetic education curricula for non-specialist students.

## 1 Introduction

Music aesthetic literacy, as a core component of individual cultural perception and artistic expression, represents a critical goal of quality education in the new era. The 2018 Chinese National Education Conference explicitly emphasized the strategy of “cultivating people through aesthetics and culture,” advocating for the enhancement of students’ comprehensive literacy through aesthetic education, with particular attention to the artistic development of non-music major students (Eryong and Li, 2020). In higher education, music aesthetic education for non-music majors serves not only as a vehicle for cultural inheritance but also as a vital pathway for fostering innovative thinking and sound personalities (Ren, 2021). However, research targeting this specific group remains limited, especially regarding how classroom environments systematically influence the formation of their aesthetic literacy.

Classroom climate, as the microecological environment of teaching and learning, encompasses core dimensions such as physical environment (PE), teacher–student interaction (TSI), peer relationships (PR), and teachers’ orientation toward learning (TOTL), exerting profound effects on students’ cognitive, emotional, and behavioral development (Konrad et al., 2024). In music education settings, a positive classroom climate not only stimulates learning motivation but also enhances students’ aesthetic perception through immersive artistic experiences (Papageorgi and Economidou Stavrou, 2023; Hendricks et al., 2023). For example, high-quality teacher–student interactions can deepen students’ emotional resonance with musical works, while a well-designed physical environment (e.g., audio equipment, spatial layout) provides the material foundation for aesthetic experiences (Jiang and Cheong, 2023; Liang, 2023). However, existing studies have confirmed the positive effects of classroom climate on academic achievement and psychological well-being (Zhao et al., 2025a,b); its specific role in cultivating musical aesthetic literacy, especially among non-music majors, warrants further exploration.

Self-efficacy, defined as an individual’s belief in their ability to execute behaviors to achieve specific outcomes, serves as both an outcome and a mediating variable in educational processes (Bandura, 2001). In music learning, students with higher self-efficacy are more likely to engage proactively in aesthetic practices, thereby deepening their understanding of musical connotations (Esteve-Faubel et al., 2021; Taş and Atılgan, 2022). However, most existing research focuses on the direct effects of self-efficacy on musical skill acquisition, neglecting its mediating role in the relationship between classroom climate and aesthetic literacy. This is particularly true for how different classroom climate dimensions (e.g., TSI vs. TOTL) exert differential effects through self-efficacy. For non-music majors, who often lack systematic musical training, this mediating mechanism may be even more critical—their reliance on classroom environments is stronger, and self-efficacy enhancement could become a key bridge to overcoming bottlenecks in aesthetic development (Chen, 2024).

Music aesthetic education in Chinese universities faces unique challenges: a large population of non-music majors and general education–oriented curricula necessitate optimizing classroom environments to improve educational effectiveness (Jiang et al., 2024; Ren, 2021). While some local institutions have integrated traditional music into teaching, systematic evaluations of classroom climate dimensions and their associations with aesthetic literacy remain scarce. Additionally, in higher education, practices in regions like Chongqing lack a generalizable theoretical framework for enhancing students’ self-efficacy through strategies such as teacher–student interaction and physical environment optimization to promote aesthetic literacy. Filling this gap will not only refine local theories of music education but also provide practical pathways for universities to implement the fundamental task of “fostering virtue through education.”

Against this backdrop, this study focuses on non-music major students to examine how dimensions of classroom climate influence music aesthetic literacy (MAL), with a specific emphasis on the mediating role of self-efficacy (SE). The specific research questions (RQ) are:

*RQ1*: How do physical environment, teacher–student interaction, peer relationships, and teachers’ orientation toward learning directly correlate with music aesthetic literacy among non-music major students?

*RQ2*: Does self-efficacy mediate the relationship between classroom climate and music aesthetic literacy?

*RQ3*: Are there differences in mediating effects across different classroom climate dimensions?

By decomposing the multidimensional structure of classroom climate and testing the mediating pathway of self-efficacy, this study seeks to provide theoretical evidence for reforming music general education in universities. It aims to facilitate the construction of a “trinity” model integrating “environment–belief–literacy” and to promote the practical implementation of aesthetic education in nonspecialized fields.

## 2 Theoretical framework

The current study integrates Bandura’s (2001) social cognitive theory, particularly the triadic reciprocal causation model, to investigate the relationship between classroom climate dimensions and musical aesthetic literacy (MAL) among non-music majors at a college. According to Bandura’s model, human behavior is shaped by the dynamic interplay between personal, environmental, and behavioral factors. Classroom climate, a crucial environmental factor, influences students’ learning experiences and academic outcomes (Pajares, 2006). The four dimensions of classroom climate—physical environment (PE), teacher–student interaction (TSI), peer relationships (PR), and teachers’ orientation toward learning (TOTL)—serve as independent variables that affect MAL, with self-efficacy acting as a mediating factor and gender as a moderating factor.

### 2.1 Physical environment and musical aesthetic literacy

The physical classroom environment significantly impacts students’ learning experiences and academic performance. Studies have demonstrated that a well-structured and resource-rich physical environment fosters student engagement and supports positive learning outcomes (Dörnyei and Muir, 2019). For instance, Liang (2023) found that factors such as layout, lighting, and technology positively correlate with student participation and engagement in learning activities. Additionally, Niemi et al. (2024) emphasized that the physical environment contributes to students’ lives and educational experiences. The advent of technology-enhanced classrooms has further elevated student self-efficacy and engagement, particularly among female students (Mantooth et al., 2021).

### 2.2 Teacher–student interaction and musical aesthetic literacy

Research indicates that teacher-student interaction (TSI) plays a significant role in developing musical aesthetic literacy (MAL), with the teacher’s orientation towards learning having a stronger positive impact than peer relationships in college settings (Zhao et al., 2025a,b). The dynamics of these interactions influence creativity during musical tasks, where teachers alternating between convergent (instructional) and divergent (idea-generating) behaviors can foster novel student ideas. However, excessive convergent turns may limit creativity (Kupers and Dijk, 2020). Interactive and innovative teaching methods, such as the Kodály method and intelligent technologies, have been shown to improve musical literacy and performance skills, highlighting the importance of active, engaging teacher involvement (Jiang and Lu, 2025). While modern online technologies can support music learning, studies suggest that direct teacher guidance still yields higher-quality musical knowledge acquisition than self-directed learning without teachers (Yao and Li, 2023). Furthermore, authentic collaborative experiences in music education, such as ensemble performances, enhance students’ musical understanding, aesthetic appreciation, and creative confidence through social and contextual learning (De Bruin et al., 2020). Overall, effective teacher-student interaction, combining instructional support and creative facilitation, is crucial for cultivating learners’ musical aesthetic literacy and creativity.

### 2.3 Peer relationships and musical aesthetic literacy

Peer relationships (PR) are crucial in shaping students’ social and academic experiences in the classroom. Positive peer interactions foster collaboration, communication, and shared learning, thereby enhancing students’ self-confidence and self-efficacy. Peer learning in music education has been shown to boost creativity and engagement, particularly through peer assessment and feedback (Lin, 2021). Gender also moderates peer interactions, with studies showing that female students tend to engage in deeper, more expressive forms of peer interaction in artistic settings (Ejsing-Duun and Pischetola, 2023).

### 2.4 Teachers’ orientation toward learning and musical aesthetic literacy

A teacher’s orientation toward learning (TOTL) refers to their pedagogical approach and its alignment with students’ needs and goals. Research suggests that TOTL also impacts students’ self-efficacy, with supportive teaching styles fostering greater student engagement and confidence (Filgona et al., 2020). Hypothesis 4 proposes that TOTL positively influences MAL, with self-efficacy as the mediating mechanism (Lauermann and Berger, 2021).

### 2.5 Self-efficacy as a mediating factor

**Figure 1:**
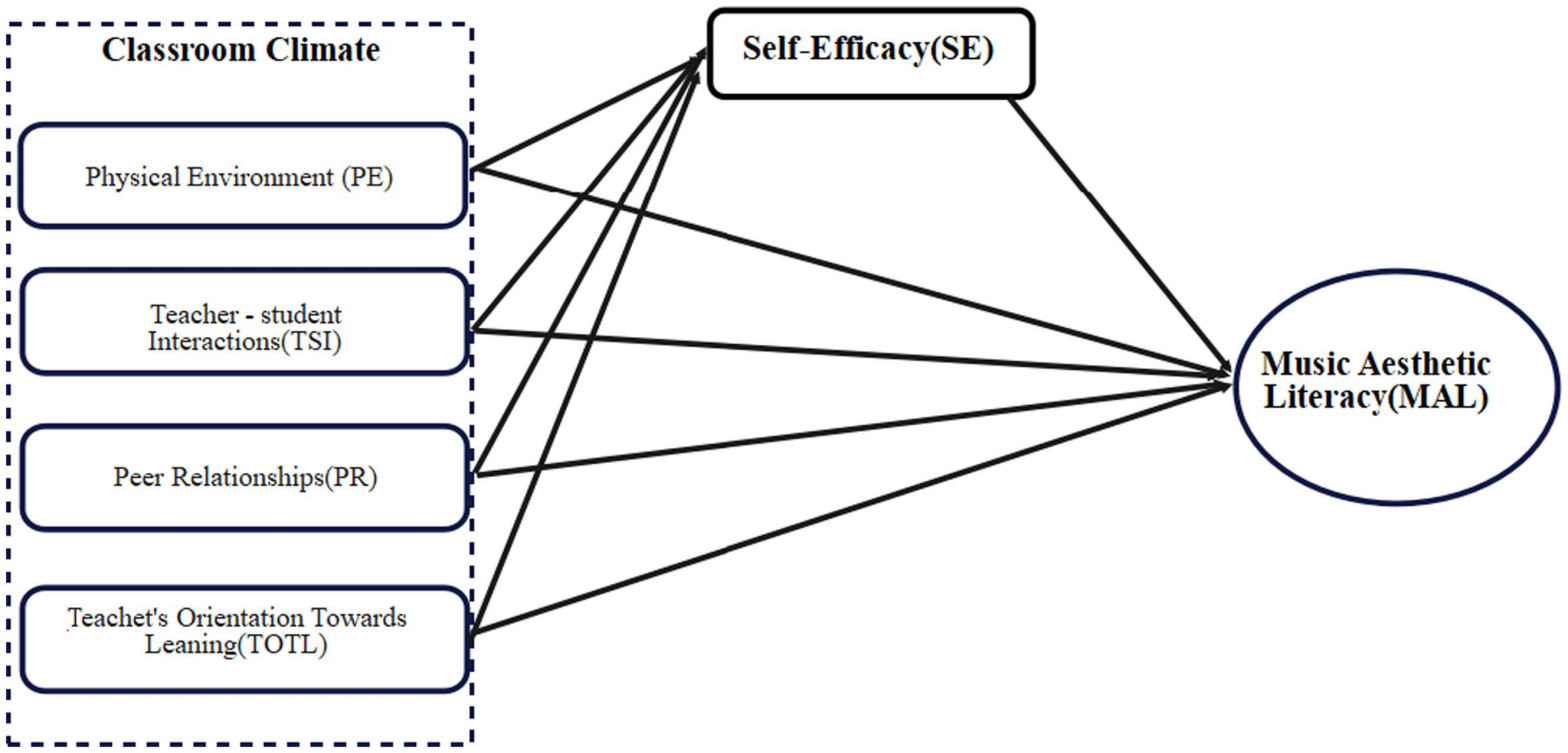
Theoretical framework.

Self-efficacy, a key personal factor in Bandura’s model, mediates the relationship between classroom climate and MAL. Higher self-efficacy is associated with increased academic performance, confidence, and resilience (Bian and Jiang, 2025; Luo et al., 2023). Previous research has shown that classroom environments, including physical space, teacher support, and peer relationships, can significantly enhance students’ self-efficacy (Khuhro, 2024). Research consistently shows that self-efficacy plays a significant mediating role in music education, influencing students’ psychological well-being, motivation, engagement, and academic performance. Studies indicate that higher self-efficacy is linked to reduced academic burnout among music education students by strengthening their professional identity and resilience (Zhu, 2025). Music education positively impacts psychological well-being, which in turn enhances academic outcomes, with self-efficacy and self-esteem serving as key mediators in this relationship (Sun, 2022). Additionally, self-efficacy mediates the effects of flow and learning motivation on learning outcomes, with prior experience strengthening these effects (Su et al., 2024). Self-efficacy also promotes sustainable engagement and academic achievement by fostering learning agility and motivation (Chen, 2024). Practical strategies to develop self-efficacy in music education include focusing on mastery experiences and verbal persuasion, which can help reduce performance anxiety and improve achievement (Gill et al., 2022; Zelenak, 2020). Consequently, the theoretical framework positions self-efficacy as a mediating factor and gender as a moderating factor in the relationship between classroom climate (comprising PE, TSI, PR, and TOTL) and musical aesthetic literacy (Figure 1). This framework aligns with Bandura’s triadic reciprocal causation model. It provides a comprehensive lens for examining how environmental and personal factors interact to shape students’ academic and aesthetic outcomes in music education.

## 3 Research methodology

### 3.1 Participants and sampling

The subjects of this study are undergraduate students from universities in Chongqing, China. Chongqing comprises 76 higher education institutions, including 28 that offer undergraduate education, 45 that offer vocational education, and three that offer adult education. This study focuses on undergraduates who are not music majors and includes 28 universities as our research subjects. Convenience sampling was utilized throughout formal testing. According to Etikan et al. (2016), this nonprobabilistic technique selects individuals of the target population based on their accessibility, availability, willingness to participate, and geographic closeness. This study selected five universities from a pool of 28 that provide undergraduate education as participants, employing cluster sampling to choose students from administrative classes for the survey. In addition to the availability of relevant courses and geographical accessibility, the sample selection was based on the following criteria to maximize the diversity and representativeness of the sample within the higher education context: (1) The five selected universities cover different types of institutions, including comprehensive universities, science and engineering-focused universities, and normal universities. This ensures that the sample can reflect the impacts of distinct academic atmospheres and campus cultures on students. (2) Consideration was given to including “Double First-Class” initiative universities, key provincial universities, and local application-oriented undergraduate universities, thereby covering institutions with varying levels of academic resources and student quality. (3) The selected universities exhibit variations in total student enrollment, which helps to incorporate student experiences across different campus scales and environments. (4) All selected institutions offer public optional courses or restrictive elective courses in music for non-music major students in compliance with the requirements for aesthetic education courses in general universities issued by China’s Ministry of Education, ensuring the consistency of the research context. Through the criteria above, we aim to obtain a sample that, while employing convenience sampling, exhibits certain heterogeneity in key institutional characteristics. This enhances the explanatory power of the research findings among undergraduate universities in Chongqing. These five universities have implemented music aesthetic education courses, and students completed them by December 1, 2024. The questionnaires were distributed across five universities, resulting in 525 surveys and 505 valid responses. The linear multiple regression analysis, with an effect size of 0.02, a probability of error of 0.05, a power level of 0.80, and five predictors, required a minimum sample size of 399, as calculated using GPower and the F test. This survey exceeded the required sample size, with 505 eligible respondents.

### 3.2 Data collection

In this study, an online questionnaire was distributed via “Questionnaire Star.” Initially, 30 university students were invited via WeChat groups to participate in a pilot test of the questionnaire. Participants in the pilot study were recruited from another comprehensive university in Chongqing that was not included in the formal research. This university shares similar characteristics with the institutions in the formal sample in terms of institutional type, student scale, and the design of aesthetic education courses. The pilot study sample was largely consistent with the subsequent formal sample on key demographic variables. All participants were non-music majors with diverse academic backgrounds, including humanities and social sciences, science and engineering, and had completed the university’s required music-related aesthetic education courses. This arrangement ensured that the pilot study could test the measurement tools in a context highly similar to that of the main research, thereby guaranteeing the validity and relevance of the pilot study results. Participants in the pilot study completed the initial version of the questionnaire. They were asked to provide written feedback on the clarity and ambiguity of the items, as well as the questionnaire’s overall length. Based on the collected data and analysis, we made the following key revisions: (1) Some participants reported that certain scale items derived from Western educational contexts (e.g., “In my class, teachers make us follow the rules and obey their orders”) involved cultural differences in understanding. To address this, we adjusted the expressions to be more consistent with the classroom context of Chinese universities, for example, revising the item above to “In class, teachers require us to abide by course discipline.” (2) In the Music Aesthetic Literacy Scale, some participants pointed out that the expression “the principles of artistic forms in folk music” was overly academic. We simplified it to “I understand the basic artistic characteristics of folk music” to improve the item’s comprehensibility. The final version of the questionnaire was disseminated in student WeChat groups by university instructors or displayed on classroom slides during lectures, inviting students to participate in the survey. Participation was voluntary for all invited students. The online questionnaire remained accessible from December 1, 2024, to December 30, 2024, resulting in a total of 505 valid responses. Table 1 presents basic information about the sample. This study complied with all relevant ethical norms and regulations to guarantee the protection of participants’ informed consent, privacy, and data confidentiality. The research methodologies and protocols received approval from the necessary ethics committee to guarantee ethical adherence and safeguard participants’ rights.

**Table 1:** Demographic frequency and number analysis.

Demographic attributes	Total	Percentage
Gender		
Male	243	48.12
Female	262	51.88
Qualification		
Sophomore	214	42.38
Junior	239	47.32
Senior	52	10.30
Postgraduate	0	0.00
Professional category		
Humanities and social sciences	164	32.48
Science and engineering	153	30.3
Art (without music)	188	37.23
Music family background		
Yes	264	52.28
No	241	47.72

### 3.3 Measurement and validity

This study employed three validated scales to measure classroom climate factors, self-efficacy, and music aesthetic literacy (MAL). All items were rated on a 5-point Likert scale. To ensure conceptual equivalence and cultural appropriateness in the Chinese higher education context, all scales underwent a systematic cross-cultural adaptation procedure that included back-translation following Brislin’s (1970) model, expert review, and pretesting.

#### 3.3.1 Classroom climate scale

The four independent variables—physical environment (PE, six items), teacher–student interaction (TSI, eight items), peer relationships (PR, five items), and teachers’ orientation toward learning (TOTL, five items)—were adapted from the Classroom Climate Scale developed by López et al. (2018). To ensure the conceptual equivalence, cultural adaptability, and measurement validity of all Western-derived scales in the context of Chinese higher education, we adopted Brislin’s (1970) classic back-translation model, supplemented by expert review and pretesting, to conduct a systematic cross-cultural adaptation. The research team compared and discussed two Chinese translations, resolved ambiguities and discrepancies in the translation process, and developed a consensus-based initial Chinese version. Items with unclear expressions, cultural conflicts, or poor comprehensibility were revised and refined to form the pretest version. Originally designed to assess elementary students’ perceptions of classroom climate, the scale was modified for college students by adjusting terminology and simplifying language to align with higher education contexts. Example items include:

PE: “We can hear the teacher and other classmates clearly from anywhere in the classroom.”

TSI: “In my class, teachers make us follow the rules and obey their orders.”

PR: “During class, we can talk and participate without being teased or insulted by our classmates.”

TOTL: “Our teachers tell us that we can all learn, even if it is at a different pace.”

The original scale demonstrated strong reliability, with a total Cronbach’s *α* of 0.93 and subscale α values ranging from 0.75 (PE) to 0.86 (PR). In this study, minor linguistic adaptations were made to enhance contextual appropriateness, but the core constructs remained intact.

#### 3.3.2 Self-efficacy scale

Self-efficacy was measured using the 10-item scale developed by Luo et al. (2023), which focuses on students’ confidence in overcoming academic challenges. Example items include: “I am convinced that I am able to successfully learn all relevant subject content even if it is difficult.” “When I try really hard, I am able to learn even the most difficult content.” The scale exhibited excellent reliability in its original form (*α* = 0.928). For this study, the wording was slightly adjusted to align with music learning contexts.

#### 3.3.3 Music aesthetic literacy (MAL) scale

The MAL scale was adapted from Wu’s (2017) Aesthetic Literacy Scale, originally developed in a Chinese cultural context. A key characteristic of this adapted scale is its cultural specificity, as it is designed to measure music aesthetic literacy grounded in the Chinese artistic and educational environment. To focus on music-specific literacy, the “visual expression and appreciation skills” subscale was removed from the original five-dimension scale, and the wording was revised to be music-specific (e.g., “artistic attitude” was changed to “musical attitude”).

The final scale included four dimensions, with item content reflecting both universal aspects of music engagement and elements specific to Chinese musical culture:

Habit (e.g., “I often visit music performances”).
Attitude (e.g., “I think music is good for life”).
Knowledge (e.g., “I know the categories of Chinese national instrumental music”).
Performance and Appreciation Skill (e.g., “I understand the principle of the art form of folk music”).

The original scale demonstrated high reliability (*α* = 0.94). In our study, the culturally and contextually adapted MAL scale showed excellent internal consistency in a pilot test (*n* = 50, *α* = 0.91), supporting its use for the present sample.

#### 3.3.4 Reliability and validity test

To ensure the psychometric robustness of the measurement scales, a comprehensive reliability and validity assessment was conducted.

Prior to the analysis, the Kaiser-Meyer-Olkin (KMO) measure of sampling adequacy and Bartlett’s test of sphericity were examined. The results indicated a KMO value of 0.946, significantly exceeding the recommended threshold of 0.6, and Bartlett’s test was statistically significant (*χ*^2^ = 19001.097, *p* < 0.001). These results confirm that the data are highly suitable for factor analysis and that the item correlations are sufficient to proceed. The measurement model was evaluated for internal consistency reliability and convergent validity. As presented in Table 2, all constructs demonstrated excellent reliability. Cronbach’s alpha coefficients ranged from 0.751 to 0.919, and composite reliability (CR) values ranged from 0.842 to 0.934, all surpassing the conservative threshold of 0.70. This indicates a high level of internal consistency among the items measuring each construct. Furthermore, convergent validity was established, as the average variance extracted (AVE) for each construct exceeded the minimum required of 0.50, ranging from 0.572 to 0.681. This confirms that the items converge well to represent their intended latent constructs.

**Table 2:** Reliability and convergent validity of constructs.

Construct	Cronbach’s alpha	Composite reliability (CR)	Average variance extracted (AVE)
Music aesthetic literacy (MAL)	0.751	0.842	0.572
Physical environment (PE)	0.905	0.927	0.679
Peer relationships (PR)	0.861	0.900	0.643
Self-efficacy (SE)	0.919	0.934	0.638
Teachers’ orientation (TOTL)	0.883	0.914	0.681

Discriminant validity, which assesses the extent to which a construct is distinct from others, was verified using two rigorous criteria. First, the Fornell–Larcker criterion was applied. As shown in Table 3, the square root of the AVE (shown on the diagonal) for each construct was greater than its highest correlation with any other construct (off-diagonal values), confirming that each construct shares more variance with its own indicators than with others.

**Table 3:** Discriminant validity: Fornell–Larcker criterion.

Construct	MAL	PE	PR	SE	TOTL	TSI
MAL	**0.756**					
PE	0.453	**0.824**				
PR	0.426	0.414	**0.802**			
SE	0.558	0.465	0.445	**0.799**		
TOTL	0.521	0.380	0.358	0.487	**0.825**	
TSI	0.628	0.395	0.396	0.546	0.429	**0.778**

Diagonals (in bold) represent the square root of the AVE.

Second, the Heterotrait-Monotrait (HTMT) ratio of correlations was examined. All HTMT values, as displayed in Table 4, were below the stringent threshold of 0.85, providing further strong evidence of discriminant validity. Additionally, the cross-loadings matrix confirmed that all indicator loadings were highest on their respective constructs, with no significant cross-loadings observed.

**Table 4:** Discriminant validity: HTMT criterion.

Construct	MAL	PE	PR	SE	TOTL	TSI
MAL						
PE	0.543					
PR	0.527	0.466				
SE	0.666	0.508	0.499			
TOTL	0.633	0.424	0.407	0.536		
TSI	0.754	0.434	0.447	0.594	0.476	

Finally, the model’s predictive relevance was assessed using the Stone–Geisser *Q*^2^ value obtained via a blindfolding procedure. The *Q*^2^ values for the key endogenous constructs were 0.299 for MAL and 0.277 for SE. As these values are substantially greater than zero, they indicate that the model posits sufficient predictive relevance for these variables.

In summary, the results from the comprehensive reliability and validity analysis confirm that all measurement scales possess strong psychometric properties, ensuring the robustness and credibility of subsequent path analysis and hypothesis testing.

### 3.4 Data analysis

Partial least squares structural equation modeling (PLS-SEM) is particularly suitable for testing theoretical frameworks from a predictive perspective. This study employs PLS-SEM as the primary method of data analysis, using SmartPLS 4.0. This approach was selected because it is effective for complex models involving multiple latent variables and is well-suited for examining both mediation and moderation effects (Hair et al., 2019).

#### 3.4.1 Description analysis

Conduct descriptive statistics on all observed variables of the Classroom Climate Scale and Music Aesthetic Literacy Scale, calculating the mean, standard deviation (SD), skewness, and kurtosis for each variable. The mean reflects the average level of a variable, while the standard deviation measures the degree of data dispersion. Skewness and kurtosis are used to assess the normality of the data (absolute values less than 1 indicate approximate normality). Statistical calculations were performed using IBM SPSS 27.0 software to provide basic information on data distribution for subsequent analysis.

#### 3.4.2 Correlation analysis

Pearson correlation coefficients were used to examine the linear correlation between each dimension of classroom climate and each dimension of music aesthetic literacy. Taking the four dimensions of classroom climate (PE, PR, TOTL, TSI) as independent variables and music aesthetic literacy (MAL) as the dependent variable, calculate the correlation coefficients between each pair of variables. A two-tailed test was employed with a significance level of *α* = 0.05, focusing on correlations significant at *p* < 0.05 and *p* < 0.01. The correlation coefficients and significance levels between variables are reported in a correlation matrix table, along with annotations on the direction of correlation (positive/negative) and the strength of correlation (*r* < 0.3 for weak correlation, 0.3 ≤ *r* < 0.5 for moderate correlation, and *r* ≥ 0.5 for strong correlation).

#### 3.4.3 Mediation analysis

To test the mediating effects of self-efficacy, the indirect effects of classroom climate factors—physical environment, teacher–student interaction, peer relationships, and teachers’ learning orientation—on the acquisition of music aesthetic literacy were evaluated. Self-efficacy is introduced as the mediating variable, and the significance of mediation is assessed using bootstrapping with 5,000 subsamples. This nonparametric procedure provides more robust estimates of indirect effects and confidence intervals. To assess the strength of the mediation effects, the specific indirect effect coefficients were calculated for each hypothesized path. The analysis focused on the significance and magnitude of these indirect paths to determine whether self-efficacy significantly mediates the relationship between each classroom climate factor and music aesthetic literacy.

## 4 Results

This section presents the study’s findings, which investigated the relationships among classroom climate dimensions, self-efficacy, and music aesthetic literacy among Chinese non-music-major college students. The results are organized into four parts: descriptive statistics and correlations, assessment of the measurement model, direct effects testing, and mediation analysis.

### 4.1 Descriptive statistics and correlational analysis

Prior to testing the structural model, descriptive statistics and bivariate correlations were computed for all key variables. As detailed in Table 5, all variables demonstrated means above the midpoint of the scale, with standard deviations indicating adequate variability. Skewness and kurtosis values for all constructs fell within the acceptable range of ±1, suggesting that the data approximated a normal distribution (Cain et al., 2017).

**Table 5:** Means, standard deviations, and correlations among key variables (*N* = 505).

Variable	M	SD	1	2	3	4	5	6
1. PE	3.85	0.71	—					
2. TSI	3.92	0.68	0.562***	—				
3. PR	3.96	0.74	0.501***	0.588***	—			
4. TOTL	4.02	0.66	0.523***	0.621***	0.594***	—		
5. SE	3.78	0.73	0.463***	0.579***	0.488***	0.532***	—	
6. MAL	3.88	0.69	0.512***	0.626***	0.554***	0.609***	0.515***	—

PE, physical environment; TSI, teacher-student interaction; PR, peer relationships; TOTL, teachers’ orientation toward learning; SE, self-efficacy; MAL, music aesthetic literacy. ***p < 0.001.

Pearson correlation analysis revealed significant positive correlations among all classroom climate dimensions, self-efficacy (SE), and music aesthetic literacy (MAL). All correlations were significant at *p* < 0.001. As shown in Table 2, teacher-student interaction (TSI) exhibited the strongest bivariate correlation with MAL (*r* = 0.626), followed by teachers’ orientation toward learning (TOTL, *r* = 0.609), peer relationships (PR, *r* = 0.554), and physical environment (PE, *r* = 0.512). Self-efficacy was also significantly correlated with all classroom climate dimensions and with MAL (*r* = 0.515). These preliminary results provided initial support for proceeding with the proposed mediation analysis.

### 4.2 Assessment of the measurement model

The measurement model was evaluated for reliability and validity before testing the structural relationships. The measurement model demonstrated acceptable fit to the data based on the following approximate indices: *χ*^2^/*df* = 2.38, CFI = 0.936, TLI = 0.928, RMSEA = 0.052, SRMR = 0.043. All factor loadings were significant and exceeded 0.60. Composite reliability (CR) values for all constructs ranged from 0.879 to 0.949, exceeding the recommended threshold of 0.70, thus confirming internal consistency. The average variance extracted (AVE) for each construct ranged from 0.548 to 0.745, all above 0.50, establishing convergent validity. Furthermore, discriminant validity was confirmed, as the square root of each construct’s AVE was greater than its correlations with other constructs (Fornell and Larcker, 1981).

### 4.3 Direct effects and mediating role of self-efficacy

To test the hypothesized direct and mediating effects, Partial Least Squares Structural Equation Modeling (PLS-SEM) was employed, with a bootstrapping procedure of 5,000 resamples. The model explained a substantial proportion of the variance in both self-efficacy (*R*^2^ = 0.439) and music aesthetic literacy (*R*^2^ = 0.553).

#### 4.3.1 Direct effects on music aesthetic literacy

The analysis of direct paths revealed that all four classroom climate dimensions had significant positive direct effects on MAL, controlling for the mediator. As summarized in Table 3, teachers’ orientation toward learning (TOTL) demonstrated the strongest direct effect on MAL (*β* = 0.264, *p* < 0.001), followed by peer relationships (PR, *β* = 0.189, *p* = 0.007). The direct effects of physical environment (PE, *β* = 0.163, *p* = 0.010) and teacher-student interaction (TSI, *β* = 0.164, *p* = 0.003) were also significant and positive. Furthermore, self-efficacy (SE) had a significant direct effect on MAL (*β* = 0.153, *p* = 0.003).

#### 4.3.2 Mediating effects of self-efficacy

The mediation analysis tested the indirect effects of the classroom climate dimensions on MAL through self-efficacy. As presented in Table 6, the bootstrapping results indicated that all four indirect paths were statistically significant.

**Table 6:** Direct, indirect effects, and model fit indices.

Path	Direct effect (*β*)	*p*-value	Indirect effect (*β*)	*p*-value	95% CI for indirect effect	Total effect (*β*)
PE → MAL	0.163	0.010	0.029	0.017	[0.008, 0.057]	0.192
TSI → MAL	0.164	0.003	0.047	0.005	[0.015, 0.084]	0.211
PR → MAL	0.189	0.007	0.025	0.025	[0.005, 0.051]	0.214
TOTL → MAL	0.264	<0.001	0.034	0.020	[0.008, 0.067]	0.298
SE → MAL	0.153	0.003	—	—	—	—
PE → SE	0.190	<0.001	—	—	—	—
TSI → SE	0.310	<0.001	—	—	—	—
PR → SE	0.164	<0.001	—	—	—	—
TOTL → SE	0.223	<0.001	—	—	—	—

Model fit indices						
*R*^2^ (MAL)	*R*^2^ (SE)	*χ*^2^/*df*	CFI	TLI	RMSEA	SRMR
0.553	0.439	2.38	0.936	0.928	0.052	0.043

TSI → SE → MAL: *β* = 0.047, 95% CI [0.015, 0.084], *p* = 0.005.

TOTL → SE → MAL: *β* = 0.034, 95% CI [0.008, 0.067], *p* = 0.020.

PE → SE → MAL: *β* = 0.029, 95% CI [0.008, 0.057], *p* = 0.017.

PR → SE → MAL: *β* = 0.025, 95% CI [0.005, 0.051], *p* = 0.025.

Given that both the direct effects of the climate dimensions on MAL and the indirect effects through SE were significant, these results support a partial mediation model (Baron and Kenny, 1986). Self-efficacy partially explains the relationship between each dimension of classroom climate and music aesthetic literacy. Among the dimensions, TSI showed the strongest total effect on SE (*β* = 0.310, *p* < 0.001) and, consequently, the strongest indirect effect on MAL.

### 4.4 Effect size (*f*^2^) and moderating effects

The effect sizes (*f*^2^) of the predictors were examined to assess their substantive impact. For the dependent variable MAL, TOTL had the largest effect size among the predictors, though its absolute magnitude was small (*f*^2^ = 0.055), followed by the interaction term Gender x TSI (*f*^2^ = 0.064). For the mediator SE, TSI demonstrated the largest effect size (*f*^2^ = 0.122), indicating its strong influence on self-efficacy.

Regarding moderation, the analysis revealed a significant interaction between gender and TSI on MAL (*β* = 0.403, *p* < 0.001), suggesting that the effect of teacher-student interaction on aesthetic literacy differs for male and female students. The interaction between gender and PR was also significant (*β* = −0.188, *p* = 0.027). However, the interactions between gender and PE (*β* = −0.106, *p* = 0.168) and between gender and TOTL (*β* = −0.077, *p* = 0.343) were not statistically significant.

## 5 Discussion

This study investigated the mechanisms by which classroom climate dimensions influence music aesthetic literacy (MAL) among Chinese non-music-major university students, with a specific focus on the mediating role of self-efficacy (SE). The findings robustly support our hypothesized model, revealing that a positive classroom climate not only directly fosters MAL but also does so indirectly by bolstering students’ confidence in their learning capabilities. The results provide clear answers to our research questions. In response to RQ1, all four dimensions of classroom climate—physical environment (PE), teacher-student interaction (TSI), peer relationships (PR), and teachers’ orientation toward learning (TOTL)—demonstrated significant positive direct effects on MAL. This indicates that a supportive learning environment, characterized by adequate resources, positive interactions, collaborative peers, and a growth-oriented teacher, directly enhances students’ aesthetic habits, attitudes, knowledge, and skills. Notably, TOTL emerged as the strongest direct predictor, underscoring the paramount importance of the teacher’s pedagogical philosophy. Addressing RQ2, the mediation analysis confirmed that self-efficacy serves as a significant partial mediator in the relationships between all classroom climate dimensions and MAL. The significant indirect effects reveal that a positive classroom climate builds students’ belief in their ability to understand and engage with music, which in turn empowers them to develop higher levels of aesthetic literacy. Finally, concerning RQ3, the findings confirm that the strength of these direct and mediating pathways varies across dimensions. For instance, while TOTL had the strongest direct effect on MAL, TSI exerted the strongest total influence on self-efficacy and, consequently, the largest indirect effect on MAL. Furthermore, gender was found to moderate the effects of TSI and PR on MAL, suggesting that the impact of classroom social interactions may differ for male and female students. The strong direct effect of TOTL may stem from Chinese students’ high sensitivity to teachers’ authority and expectations; the robust predictive power of TSI on SE may reflect that in non-specialized fields, students are particularly reliant on teachers’ immediate feedback and encouragement to build confidence.

Our findings offer compelling empirical support for Bandura’s (2001) triadic reciprocal causation model within the domain of music aesthetic education. The model posits a dynamic interplay between environmental, personal, and behavioral factors. The significant direct effects of all classroom climate dimensions on MAL powerfully illustrate how the environment directly shapes behavior (the development of literacy). A well-equipped physical environment (PE) facilitates immersive aesthetic experiences, as suggested by Liang (2023), while positive teacher-student interactions (TSI) provide the guidance and emotional support necessary for deep artistic engagement (Kupers and Dijk, 2020; Zhao et al., 2025a,b). Supportive peer relationships (PR) create a safe space for aesthetic exploration and expression (Lin, 2021), and a teacher’s growth-oriented beliefs (TOTL) directly model and encourage a positive and persistent attitude toward musical learning (Lauermann and Berger, 2021). The documented mediating role of self-efficacy elucidates the second half of this triad: how the environment influences personal factors, which, in turn, drive behavior. Our results show that a supportive classroom environment is a potent source of efficacy information. Encouraging feedback from teachers (TSI), observing peers succeed (PR), having access to quality learning tools (PE), and being in a class where the teacher believes everyone can learn (TOTL) all serve as mastery experiences, vicarious experiences, and verbal persuasion—the primary sources of self-efficacy outlined by Bandura (2001). This enhanced self-efficacy, as demonstrated in prior research (Chen, 2024; Zelenak, 2020), then motivates students to engage more proactively, persist through challenges, and ultimately achieve a higher level of music aesthetic literacy.

The nuanced differences in the pathways align with and extend previous literature. The finding that TOTL had the strongest direct effect on MAL highlights that for non-music majors, the teacher’s fundamental belief in their capacity to learn may be even more critical than specific instructional techniques. This growth mindset, communicated by the teacher, may directly reduce anxiety and foster the open, receptive attitude essential for aesthetic appreciation (Esteve-Faubel et al., 2021). Conversely, TSI was the strongest predictor of self-efficacy. This underscores the teacher’s role as the most influential social model and persuader in the classroom. The quality of interaction—ranging from constructive feedback to motivational support—is a primary conduit for building students’ confidence, a finding consistent with studies on teacher influence on SE (Gill et al., 2022; Khuhro, 2024). The significant moderating effect of gender on the TSI-MAL relationship further suggests that the dynamics of these interactions are complex and may be interpreted differently by students’ gender, warranting further qualitative investigation. The significant, though comparatively smaller, effects of PR and PE affirm their role as foundational elements. Peer relationships provide a community of practice where students can test their understanding and build confidence through collaboration (De Bruin et al., 2020), while the physical environment serves as the essential, albeit sometimes overlooked, material base that enables or constrains high-quality aesthetic experiences (Jiang and Cheong, 2023).

This study makes several key theoretical contributions. First, it moves beyond a monolithic view of classroom climate by deconstructing it into distinct dimensions and demonstrating how these dimensions are interconnected in fostering aesthetic outcomes. Second, it bridges a critical gap in the literature by empirically validating the mediating mechanism of self-efficacy, thereby providing a mechanistic explanation for how classroom environments translate into enhanced literacy. This elucidates the “environment-belief-literacy” model proposed in the introduction. Finally, it successfully adapts and applies Bandura’s social cognitive theory to the under-researched context of non-specialist music aesthetic education in China, demonstrating the theory’s cross-cultural and cross-disciplinary relevance.

Based on the empirical findings, this study proposes a dual-pathway approach for teaching and educational policy: for instructors, it is imperative to consciously foster a growth-oriented learning environment by emphasizing that aesthetic literacy is developable, while prioritizing supportive teacher-student interactions and collaborative peer activities to build students’ self-efficacy directly; for university administrators and policymakers, this necessitates integrating teacher training on motivational communication and classroom climate building, mandating the allocation of resources for high-quality physical-sonic environments in music classrooms, and designing curricula that systematically embed confidence-building through progressive challenges, thereby translating the evidenced “environment-belief-literacy” mechanism into concrete practices that enhance the effectiveness of aesthetic education for non-specialist students.

## 6 Conclusion

This study provides empirical evidence for the “classroom climate – self-efficacy – music aesthetic literacy” model among Chinese non-music major students. Theoretically, this study contributes to the literature by deconstructing the multidimensionality of classroom climate and empirically validating the mediating mechanism of self-efficacy within Bandura’s social cognitive framework, particularly in the under-explored context of aesthetic education for non-specialists. Several limitations temper the findings. First, the use of adapted scales, though necessary for cultural relevance, may affect measurement invariance and cross-study comparisons. Second, reliance on self-reported data risks social desirability bias and common-method bias. Third, the culturally homogeneous sample from one Chinese municipality limits generalizability to other populations. Future research should employ longitudinal designs, incorporate objective measures, and validate findings in diverse cultural contexts. Despite these limitations, this study underscores the importance of fostering supportive classroom environments, particularly through quality teacher-student interactions, to enhance students’ self-efficacy and aesthetic literacy.

## Data Availability

The raw data supporting the conclusions of this article will be made available by the authors, without undue reservation.
